# Mechanistic Pathways Linking Cannabidiol, Hemp Seed Oil and Black Sesame Oil in Hyperarousal Insomnia: A Narrative Review

**DOI:** 10.3390/clockssleep8020016

**Published:** 2026-03-31

**Authors:** Piphat Kovitkanit, Thavatchai Kamoltham

**Affiliations:** College of Health Science, Suan Sunandha Rajabhat University, Samut Songkhram 75000, Thailand; s66584955005@ssru.ac.th

**Keywords:** insomnia, hyperarousal, sleep–wake stability, neuroinflammation, endocannabinoid system, polyunsaturated fatty acids (PUFAs), cannabidiol (CBD), hemp seed oil, black sesame oil

## Abstract

Insomnia is increasingly recognized as a manifestation of multisystem dysregulation characterized by sustained physiological hyperarousal. This review situates insomnia within a framework of reciprocal disturbances across neuroendocrine, inflammatory, and autonomic pathways. It examines the potential roles of cannabidiol (CBD), polyunsaturated fatty acids (PUFAs) derived from hemp seed oil (HSO), and lignans from black sesame oil (BSO) as modulators of upstream biological processes relevant to sleep regulation. Rather than acting as direct hypnotics, these compounds are considered for their capacity to influence convergent mechanisms involved in sleep–wake stability. Preclinical evidence suggests that CBD modulates endocannabinoid and serotonergic signaling, potentially contributing to the regulation of physiological processes associated with hyperarousal. Concurrently, HSO-derived fatty acids support mitochondrial function and lipid-mediated resolution. Sesame lignans further contribute through antioxidant properties linked to redox balance, neurometabolic stability, and modulation of neural excitability. However, the current evidence base is predominantly preclinical, and definitive conclusions regarding therapeutic efficacy or optimal dosing in humans remain limited. Future research should prioritize integrative clinical studies that link these specific biological modulations to standardized sleep outcomes to determine their real-world applicability. Nevertheless, the pathways discussed align with biological domains consistently implicated in established insomnia phenotypes. This review integrates these compounds within a shared hyperarousal framework to highlight convergent upstream mechanisms that extend beyond their individual effects.

## 1. Introduction

Poor sleep has wide-ranging physiological, neurological, and psychological consequences, motivating investigation into the upstream biological pathways underlying disturbances in sleep regulation [1,2,3]. Extensive neurobiological and clinical research has demonstrated strong associations between sleep disruption and heightened systemic inflammation, which is linked to increased vulnerability to chronic disease [3,4,5]. The consequences of insufficient sleep also extend into psychological and occupational domains, where disrupted sleep has been associated with reduced emotional resilience, increased depression risk, and errors in high-stakes professional environments [6,7,8].

Insomnia, one of the most pervasive of these disturbances, is characterized by persistent deficits in sleep initiation, maintenance, or restorative quality [9,10,11]. Modern clinical frameworks increasingly conceptualize insomnia as a systemic disorder marked by sustained hyperarousal across neural, endocrine, and autonomic domains, promoting a self-perpetuating cycle of sleep fragmentation and heightened stress sensitivity [10,12,13,14]. Over time, these alterations impair executive functions, including memory processing and emotion regulation, further illustrating the condition’s broader functional burden [7,9,11]. Recent international sleep medicine guidelines continue to emphasize the clinical significance of insomnia as a prevalent and burdensome condition requiring evidence-based management [15].

Driven by the need for more targeted interventions, research attention has shifted toward identifying upstream biological pathways that precipitate sleep instability [16]. Additionally, the complex bidirectional signaling between sleep regulation and immune function suggests that modulating inflammatory and neurochemical pathways may offer significant therapeutic leverage [3,4,5,16].

Based on this rationale, cannabidiol (CBD), polyunsaturated fatty acids (PUFAs) from hemp seed oil, and lignans from black sesame oil were selected because their bioactive properties converge on upstream mechanisms implicated in hyperarousal-based insomnia [10,11,12,13,14]. In addition, they represent three distinct biochemical classes—cannabinoids, polyunsaturated fatty acids, and lignans—each influencing complementary systems that govern neuroimmune signaling, metabolic regulation, oxidative balance, and stress-responsive neural circuitry relevant to sleep–wake stability. These compounds do not act as direct hypnotics; instead, they influence multiple regulatory systems that shape susceptibility to sleep disruption. These include excitatory–inhibitory balance, stress responsivity, neuroimmune signaling, oxidative resilience, and circadian–metabolic coordination [3,4,5,16,17,18,19]. Within a unified mechanistic framework, examining these agents together helps clarify how convergent biological influences may contribute to sleep–wake stability under sustained physiological hyperarousal [12,16,19].

Public and commercial interest in these compounds has expanded in recent years. However, the underlying scientific evidence remains uneven and insufficiently integrated across studies, limiting a comprehensive understanding of how their collective interactions with upstream biological drivers of sleep–wake stability.

In light of these gaps, this review brings together mechanistic evidence from neurobiological, inflammatory, metabolic, and circadian research to examine how CBD, PUFAs, and lignans interact within established sleep-regulatory networks. Situating these mechanisms within a broader sleep-science context may help guide future translational research and hypothesis-driven clinical studies.

## 2. Literature Search Strategy

This narrative review employed a targeted literature search to synthesize mechanistic evidence on biological pathways relevant to sleep–wake regulation. These pathways describe how cannabidiol (CBD), hemp seed oil-derived polyunsaturated fatty acids (PUFAs), and black sesame oil-derived lignans may influence sleep–wake processes. A narrative approach was selected because the relevant literature spans heterogeneous domains, including molecular pharmacology, neurobiology, immunology, metabolism, and circadian biology, which limits the suitability of formal systematic reviews or quantitative meta-analyses.

Accordingly, the evidence base comprised in vitro studies, animal models, mechanistic human investigations, and integrative conceptual reviews. These sources were interpreted qualitatively to elucidate biological relevance across regulatory systems, rather than to aggregate effect sizes or formally evaluate clinical efficacy.

Literature searches were conducted using PubMed, Scopus, and Google Scholar, covering publications from January 2000 to October 2025. PubMed was used to identify core biomedical and clinically indexed literature relevant to sleep physiology, inflammation, and neurobiological mechanisms, while Scopus was selected for its broader multidisciplinary coverage, including cross-disciplinary and mechanistic studies spanning molecular pharmacology, neurobiology, immunology, metabolism, and circadian biology. This complementary approach enabled comprehensive coverage of heterogeneous evidence across multiple domains. Search terms were applied in various combinations and included “cannabidiol,” “CBD,” “endocannabinoid system,” “FAAH inhibition,” “hemp seed oil,” “polyunsaturated fatty acids,” “linoleic acid,” “alpha-linolenic acid,” “black sesame oil,” “lignans,” “sesamin,” “sesamolin,” “sleep regulation,” “insomnia,” “circadian rhythms,” “inflammation,” and “oxidative stress.”

Reference lists of seminal mechanistic studies and high-impact narrative or conceptual reviews were manually screened to ensure coverage of foundational work in sleep biology. Studies were included if they examined molecular, neural, endocrine, inflammatory, oxidative, metabolic, or circadian mechanisms relevant to sleep–wake regulation and involved CBD, hemp seed oil-derived PUFAs, or black sesame oil-derived lignans. Priority was given to studies that provided mechanistic insight, demonstrated cross-model consistency, or were relevant to hyperarousal-based models of insomnia.

Studies focused exclusively on recreational cannabis use, anecdotal reports, or content lacking mechanistic relevance to sleep–wake physiology were excluded. In keeping with established conventions for mechanistic narrative reviews, formal risk-of-bias assessments and quantitative effect-size comparisons were not performed. The primary objective was to integrate biological plausibility across experimental models rather than to establish clinical efficacy.

This strategy enabled thematic refinement of the literature and supported synthesis of convergent mechanistic pathways relevant to sleep–wake regulation, while also identifying priorities for future mechanistic and translational research.

## 3. Limitations of the Narrative Review Approach

This narrative review synthesizes mechanistic evidence across diverse biological domains relevant to sleep–wake regulation. The focus is on integrating biological plausibility from preclinical and experimental studies, rather than directly evaluating clinical efficacy or ranking interventions according to levels of evidence.

This approach supports the conceptual integration of heterogeneous findings that are not readily amenable to formal systematic review, but it may introduce selection bias. To minimize this risk, studies were selected based on mechanistic relevance, consistency across models, and alignment with established frameworks of sleep regulation and hyperarousal.

The proposed framework should therefore be viewed as hypothesis-generating and not as an evidence hierarchy or a basis for clinical recommendations. Future studies that directly link these mechanisms to standardized human sleep outcomes are required to further establish translational relevance.

## 4. Foundations of Sleep Regulation

Sleep arises from the coordinated activity of multiple biological systems that operate across different timescales. Sleep–wake stability refers to the capacity of neurobiological systems to maintain coherent transitions between wakefulness and sleep. This stability depends on the coordinated balance of excitatory and inhibitory neural activity, circadian alignment, metabolic homeostasis, and neuroimmune regulation. When these systems become disrupted, their interactions are altered, leading to dysregulation across multiple domains. This dysregulation is strongly associated with hyperarousal-based insomnia, in which persistent physiological activation interferes with normal sleep initiation and maintenance [10,13]. Two core processes—the homeostatic buildup of sleep pressure and the circadian system, governed by the suprachiasmatic nucleus (SCN)—work together to regulate the timing and consolidation of sleep [16,19,20,21,22]. Light exposure serves as the primary environmental synchronizer of circadian phase through its regulatory influence on the SCN [21,23]. During wakefulness, somnogenic substances such as adenosine accumulate, increasing the drive for sleep; at the same time, circadian timing aligns sleep with environmental light–dark cues [16].

Disruptions in these systems can impair the transition into restorative non–rapid eye movement (NREM) sleep. Reduced homeostatic sleep pressure, altered adenosinergic signaling, or circadian misalignment may interact with physiological and psychological factors to fragment sleep or increase vulnerability to arousal [20,22]. These disturbances do not occur in isolation; instead, they interact dynamically within broader neurobiological networks. A key determinant of stable sleep is the balance between inhibitory and excitatory neurotransmission. GABAergic activity promotes sleep initiation and NREM stability, whereas excessive glutamatergic or limbic activation can sustain cortical hyperarousal, a central feature of insomnia [10,11,13]. Recent analyses specifically highlight the GABAergic system as a key therapeutic target in insomnia [24]. Cognitive and emotional processes, including worry and conditioned arousal, further enhance neural sensitivity to internal and external stimuli [14].

Stress-response systems intersect with neural pathways that govern sleep–wake regulation. Heightened activation of the hypothalamic–pituitary–adrenal (HPA) axis disrupts normal cortisol rhythms, while increased sympathetic nervous system activity interferes with the physiological downshift required for sleep initiation [12,19]. Sustained hyperarousal is further associated with elevated inflammatory activity, oxidative stress, metabolic strain, and mitochondrial dysfunction, all of which contribute to less consolidated and more fragmented sleep patterns [3,5,25]. Previous studies document a bidirectional relationship between sleep and immune function, highlighting reciprocal interactions between sleep disruption and inflammatory signaling processes [26].

Because the biological systems governing sleep are interconnected, disturbances in one domain often cascade into others, amplifying the overall regulatory burden [3,4,5,12,16]. Changes in neurotransmitter balance, stress responsivity, immune signaling, metabolic regulation, or circadian timing interact across broader neurobiological networks that shape sleep–wake stability [3,4,5,12,16,19]. Consequently, sleep disruption reflects the combined effects of multiple regulatory systems rather than the failure of a single pathway [10,12,13,14].

**Figure 1:**
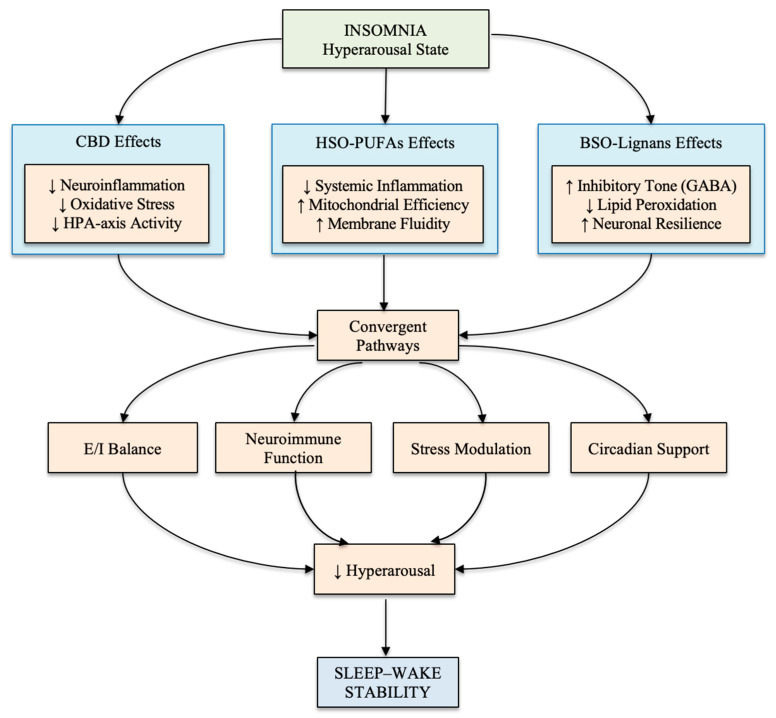
Conceptual framework illustrating convergent biological pathways through which cannabidiol (CBD), hemp seed oil-derived polyunsaturated fatty acids (PUFAs), and black sesame oil-derived lignans may influence sleep–wake regulation within a hyperarousal-based model of insomnia. These bioactive compounds interact with multiple biological domains, including neuroimmune signaling, oxidative stress regulation, stress-responsive neurocircuitry, and circadian–metabolic coordination. CBD is depicted as modulating endocannabinoid and serotonergic (5-HT1A) signaling [27,28]; PUFAs are depicted as supporting lipid-mediated inflammatory resolution and membrane homeostasis [29,30], and lignans are depicted as contributing to antioxidant and GABAergic processes [18,31].

This multisystem perspective provides a framework for examining how specific bioactive compounds may act on upstream mechanisms relevant to sleep–wake regulation, particularly within contemporary models that conceptualize insomnia as a disorder of sustained physiological hyperarousal [10,12,13,14]. It also allows assessment of whether proposed mechanistic actions intersect with established determinants of sleep regulation, including excitatory–inhibitory balance, stress-axis activity, neuroimmune signaling, and circadian–metabolic coordination [16,19,21,26]. An overview of these convergent interactions is presented in Figure 1.

Arrows indicate the direction of conceptual influence from compound-specific mechanisms to shared upstream regulatory domains (e.g., excitatory–inhibitory balance and neuroimmune function), which subsequently converge on pathways associated with attenuating physiological hyperarousal and stabilizing sleep–wake regulation. These arrows represent functional and integrative relationships among biological systems rather than direct causal pathways or clinically validated treatment effects.

## 5. Neurobiological Pathways Linking CBD and Sleep Regulation

In this review, the term “upstream regulatory mechanisms” refers to biological processes that influence sleep directly through systemic control pathways rather than acting on sleep-inducing receptors. These include neuroimmune signaling, oxidative stress regulation, metabolic homeostasis, endocannabinoid tone, and stress-axis activity. By modulating cellular and systems-level processes—such as neuronal excitability, redox balance, mitochondrial function, and neuroendocrine signaling—these processes shape physiological conditions that support sleep–wake stability. Dysregulation of these systems can alter arousal thresholds and increase vulnerability to insomnia with the hyperarousal model [3,10,13].

Cannabidiol (CBD) modulates endocannabinoid signaling through CB1 receptor-related pathways within brain networks that regulate mood, alertness, and circadian rhythms [27,28]. Systematic reviews support cannabinoid modulation processes associated with sleep–wake regulation, detailing interactions with endocannabinoid signaling, GABAergic transmission, and stress-responsive neural circuits [32,33,34,35]. This neuromodulatory influence extends to non-canonical targets, including transient receptor potential vanilloid 1 (TRPV1) and G protein-coupled receptor 55 (GPR55), which regulate nociception, excitatory–inhibitory balance, and neuroendocrine stress signaling [27,36]. While these secondary mechanisms remain under investigation, they highlight CBD’s capacity to influence multiple layers of neural control relevant to arousal regulation and neurophysiological stability [27].

The potential relevance of CBD to sleep architecture is further supported by its anti-inflammatory and antioxidant properties [17,37]. Persistent brain inflammation and overactive immune cells are linked to heightened brain arousal and dysregulated neuronal excitability [3,4,17]. In this context, CBD’s ability to modulate proinflammatory cytokine activity, regulate oxidative signaling pathways, and support neuroimmune balance provides a plausible pathway for influencing neural regulatory processes relevant to arousal control and neurophysiological stability [17,22,25]. These immune-related effects are indirect but may contribute to physiological conditions supporting sleep–wake regulation over time [37,38].

Central to this sleep-supportive profile is modulation of stress-sensitive neural circuitry, particularly within the amygdala, hippocampus, and hypothalamic–pituitary–adrenal (HPA) axis [19,28,39]. Experimental and neuroimaging evidence indicate that CBD dampens amygdala reactivity and supports more adaptive HPA-axis feedback, thereby attenuating stress-related signaling associated with sustained physiological hyperarousal [12,19,27,40]. Such attenuation of stress-related arousal is especially relevant for counteracting threat-based cognitive patterns that interfere with sleep initiation and maintenance in individuals experiencing chronic hyperarousal [40,41].

When considered together, this mechanistic profile positions CBD as a potential adjunctive approach rather than a conventional, direct-acting hypnotic, with the most significant relevance for individuals whose sleep disturbances are driven by emotional or physiological hyperactivation rather than primary circadian or structural sleep abnormalities [41,42,43].

Key molecular targets and mechanistic pathways through which cannabidiol (CBD) may modulate neurobiological processes relevant to sleep regulation are summarized in Table 1.

## 6. Mechanistic Contributions of Polyunsaturated Fatty Acids in Hemp Seed Oil

Hemp seed oil (HSO) contains polyunsaturated fatty acids (PUFAs), primarily linoleic acid (LA) and alpha-linolenic acid (ALA). These fatty acids are involved in metabolic and lipid signaling processes that contribute to systemic physiological stability [29,30,44]. The lipid constituents of HSO support metabolic homeostasis and mitochondrial function, thereby influencing biological systems associated with sleep–wake regulation [3,4]. From a mechanistic perspective, these properties provide a framework for understanding how HSO-derived PUFAs may mitigate systemic metabolic and bioenergetic stressors associated with physiological hyperarousal [46].

A fundamental driver of PUFA activity lies in the optimization of lipid metabolism and systemic homeostasis. Data from structurally similar PUFA-rich oils indicate that these lipids enhance lipid handling and alleviate metabolic strain, thereby modulating cardiometabolic processes linked to systemic physiological regulation [47,48]. Such systemic stabilization is highly relevant to insomnia, where chronic low-grade inflammation and metabolic dysregulation are consistently linked to alterations in energy balance and neurophysiological stability [12,14,20].

Beyond metabolic support, PUFAs directly orchestrate lipid-mediated inflammatory resolution processes. Experimental evidence suggests that omega-3 and omega-6 fatty acids engage with neuroinflammatory pathways to modulate cytokine production and glial cell reactivity [29,30]. This is particularly important because neuroinflammation fundamentally disrupts cellular signaling environments that influence neuronal stability, rather than acting solely through direct effects on sleep regulation [3,4]. Gamma-linolenic acid (GLA), a prominent omega-6 PUFA present in hemp seed oil, demonstrates specific anti-inflammatory properties in metabolic and inflammatory contexts, including modulation of proinflammatory mediators [30]. In addition, structurally related antioxidant compounds present in these oils provide protective effects for neuronal and vascular tissues by regulating redox balance and lipid peroxidation processes [18,47]. These antioxidant properties are essential for maintaining mitochondrial integrity and bioenergetic efficiency, which are critical for sustaining neural function under metabolic demand [29].

PUFAs may also affect neurobiological regulatory systems indirectly by supporting lipid environments associated with the endocannabinoid system (ECS) function. Given that the ECS regulates stress, mood, and arousal, its dysregulation is a primary contributor to the heightened vigilance observed in insomnia [36,40]. While cannabidiol (CBD) provides more direct receptor-level modulation, PUFAs appear to influence membrane lipid composition and precursor availability relevant to endocannabinoid signaling, thereby contributing to ECS-related homeostasis. These lipids also support the neural environment by maintaining microglial function and modulating signaling balance in neuroinflammatory processes [29]. Such actions help counteract cellular and network-level instability associated with chronic hyperarousal, rather than directly acting on sleep-promoting pathways [49].

At the cellular level, PUFAs enhance mitochondrial function, neuronal energy production, and synaptic stability [50]. These mitochondrial effects are intrinsically linked to circadian regulation, as circadian oscillators coordinate metabolic cycles that rely heavily on mitochondrial integrity [47,50]. Emerging evidence indicates that dietary PUFAs can modulate circadian clock gene expression and metabolic cycles, suggesting a role in circadian–metabolic coupling rather than direct regulation of sleep architecture [35,51]. While broader biological rhythms, including infradian cycles and environmental light exposure, may influence sleep physiology, their interactions with PUFA-mediated metabolic processes remain incompletely characterized. Accordingly, these factors should be considered as contextual modulators rather than primary mechanistic drivers within this framework.

Infradian rhythms, including menstrual and seasonal cycles, can influence neuroendocrine signaling, inflammatory activity, and metabolic processes relevant to sleep regulation. For example, fluctuations in estrogen and progesterone across the menstrual cycle have been shown to modulate GABAergic transmission and stress-related pathways associated with hyperarousal and sleep disturbance [52]. Seasonal variations may also affect metabolic and inflammatory tone, which are increasingly recognized as contributors to sleep quality and circadian stability [53]. These longer biological rhythms may therefore influence individual responses to bioactive compounds such as cannabidiol, polyunsaturated fatty acids, and lignans. However, direct evidence examining these interactions remains limited and requires further investigation.

Environmental light exposure is a key external regulator of circadian phase and plays an essential role in sleep–wake regulation. Exposure to artificial light at night can suppress melatonin secretion and delay circadian phase, thereby contributing to sleep disruption [23]. Circadian misalignment may interact with neuroendocrine, inflammatory, and metabolic pathways associated with hyperarousal insomnia. Accordingly, the effects of cannabidiol, hemp seed oil, and black sesame oil should be interpreted within the broader context of light–dark cycles. Environmental lighting conditions may significantly influence physiological responses and treatment outcomes. Future studies should account for light exposure when evaluating these interventions in relation to sleep outcomes.

Research connecting circadian misalignment to neurodegeneration further underscores how compromised neuronal resilience increases susceptibility to oxidative stress and functional decline [44,53]. Especially in the context of aging, when circadian efficiency and stress responsivity naturally deteriorate, adequate availability of PUFA may be important for maintaining the biological conditions required for healthy sleep [48,50].

From this mechanistic perspective, these pathways indicate that HSO-derived fatty acids function as systemic stabilizers. By reducing chronic inflammation and supporting metabolic and mitochondrial stability, PUFAs contribute to a neurobiological milieu associated with reduced physiological hyperarousal and improved system-level stability [14,29,50]. This positions PUFAs as upstream modulators within the multisystem framework underlying the pathophysiology of insomnia.

Future research should adopt integrative and methodologically rigorous approaches to better characterize the effects of these compounds on sleep regulation. In particular, studies should incorporate objective sleep measures such as polysomnography and actigraphy, alongside validated subjective instruments including the Pittsburgh Sleep Quality Index (PSQI). The use of multimodal biomarkers, including inflammatory cytokines, oxidative stress markers, and neuroendocrine indicators such as cortisol dynamics, may further clarify the mechanistic pathways linking physiological modulation to sleep outcomes. In addition, study designs that evaluate cross-system interactions, including neuroendocrine–inflammatory–metabolic pathways, as well as longitudinal approaches assessing temporal dynamics, will be essential to advance translational understanding.

## 7. Mechanistic Contributions of Black Sesame Oil and Lignans

The sleep-related potential of black sesame oil (BSO) stems from its unique lignan content, including sesamin and sesamolin. These compounds exhibit antioxidant, anti-inflammatory, and neuroprotective properties that contribute to cellular resilience and redox balance under conditions of physiological stress [18,46]. Preliminary clinical evidence from an elderly Thai population suggests that consumption of black sesame seeds is associated with measurable improvements in subjective sleep quality [45]. The bioactive compounds in BSO do not induce sleep directly; instead, they modulate biological systems that shape vulnerability to insomnia, including oxidative balance and redox signaling [14,18,31].

A cornerstone of sesame lignan activity is mitigating oxidative stress in neural and vascular tissues. Experimental evidence indicates that sesamin and sesamolin reduce lipid peroxidation and protect mitochondrial membranes, thereby enhancing cellular resilience under oxidative load [18]. Beyond antioxidant effects, sesamin specifically exhibits anxiolytic and neuroprotective properties in experimental models, modulating behavioral responses and protecting against neuronal excitotoxicity [31,54]. This protection is important for maintaining redox stability and mitochondrial integrity under conditions of increased metabolic demand [18].

Lignans also interact with inflammatory pathways, increasingly recognized as contributors to neural dysregulation under physiological stress. Inflammation-driven alterations in neural excitability and synaptic signaling can disrupt cellular communication and compromise network stability, rather than acting solely through direct effects on sleep processes [4,14]. By shaping inflammatory tone, BSO-derived lignans may help modulate redox-sensitive signaling pathways associated with physiological stress responses, thereby supporting biological processes relevant to neuronal function [18,31].

These lignans do not directly engage cannabinoid receptors, but their influence on lipid metabolism and oxidative balance overlaps conceptually with domains regulated by the endocannabinoid system. The ECS plays a central role in regulating arousal, emotional processing, and neural homeostasis [19,27]. Dysregulation of ECS signaling has been linked to stress-related arousal states [39]. The metabolic and antioxidant effects of BSO may therefore indirectly contribute to ECS-mediated resilience without requiring direct receptor-level interactions [18].

The functional efficacy of sesame lignans also varies across cultivars, reflecting natural differences in seed composition and lipid profiles. Such variability may result in subtle differences in antioxidant potency or metabolic effects among BSO preparations, underscoring the importance of source-specific considerations when evaluating mechanistic or translational relevance [46].

Beyond their antioxidant and anti-inflammatory actions, lignans intersect with broader lipid-mediated signaling pathways implicated in neurobiological processes associated with arousal regulation and physiological stability. Research on N-acylethanolamine metabolism highlights the role of lipid-derived mediators in nociception, inflammation, and sleep-related neurobiology [27]. Although lignans do not directly regulate these pathways, their capacity to shape oxidative and metabolic environments may influence overlapping neurobiological domains relevant to sleep–wake stability [18,45].

These mechanistic properties also carry implications for how sleep-related outcomes are assessed. Improvements in inflammatory or metabolic balance may be reflected differently depending on whether sleep is measured using polysomnography or actigraphy, consistent with contemporary frameworks that conceptualize insomnia as a multidimensional, transdiagnostic condition [8,10].

At a systemic level, the endothelial-protective and lipid-modulating effects of lignans contribute to the broader relationship between sleep and overall health. Sleep quality both influences and reflects systemic physiological stability, and lignans may support this bidirectional relationship by modulating oxidative burden and metabolic efficiency [1,48]. As with other lipid-derived bioactive compounds, potential interactions with metabolic pathways and drug metabolism warrant consideration in translational contexts [1,18].

On this basis, the mechanistic actions of black sesame lignans position BSO as an upstream modulator of biological processes relevant to sleep regulation. Rather than inducing sleep directly, these compounds contribute to physiological conditions associated with sleep–wake stability and reduced physiological hyperarousal [14,31,45].

The major mechanistic domains associated with cannabidiol (CBD), hemp seed oil-derived polyunsaturated fatty acids (PUFAs), and black sesame oil-derived lignans (BSO), based primarily on preclinical evidence, are summarized in Table 2.

## 8. Convergent Mechanisms Linking CBD, PUFAs, and Lignan Pathways to Sleep Regulation

Rather than functioning as direct sedatives, these compounds exert coordinated upstream modulatory effects on physiological systems. Cannabidiol (CBD) primarily engages endocannabinoid and serotonergic (5-HT1A) signaling, while hemp seed oil-derived PUFAs promote inflammatory resolution and membrane homeostasis, and black sesame-derived lignans provide antioxidant and GABAergic support [29,31,39]. This convergence suggests that sleep–wake regulation arises from integrated neurobiological and systemic mechanisms rather than isolated receptor-specific effects [13,16].

A critical point of convergence lies in maintaining excitatory–inhibitory (E/I) balance within central neural circuits. Within the hyperarousal model of insomnia, characterized by excessive cortical excitability and heightened autonomic activation, CBD’s modulation of serotonergic and endocannabinoid signaling may help attenuate neural hyperexcitability [10,13,14,39,40]. In parallel, the membrane-stabilizing effects of PUFAs and the GABAergic support provided by sesame lignans may further dampen activity within wake-promoting neural networks by influencing receptor microenvironment and synaptic signaling stability [29,31,54].

The bidirectional relationship between sleep and immune function represents another shared domain of convergence. Neuroinflammatory signaling can precipitate arousal and fragment sleep, while sleep disruption reciprocally amplifies inflammatory activity [3,4,5]. Through complementary actions—CBD’s anti-inflammatory effects, PUFA-derived pro-resolving lipid mediators, and the antioxidant and anti-inflammatory properties of lignans—these compounds may help restore immune–sleep homeostasis and reduce systemic stress burden, thereby supporting sleep continuity and psychological well-being [3,17,25].

Stress-regulatory systems constitute a further axis of convergence. Heightened hypothalamic–pituitary–adrenal (HPA) axis activity and sympathetic nervous system activation are central drivers of physiological hyperarousal, interfering with sleep initiation and maintenance [12,19,20]. Evidence suggests that CBD can attenuate stress-induced neuroendocrine responses. In parallel, PUFAs and lignans support metabolic, vascular, and endocrine resilience, potentially reducing stress-related physiological load and contributing indirectly to sleep–wake regulation [19,28,29,31].

Coordination between circadian and metabolic regulation represents an additional pathway through which sleep may be indirectly supported. Disruptions in circadian signaling and metabolic homeostasis contribute to sleep fragmentation and impaired quality, particularly in aging populations [21,47]. Although these compounds do not act as melatonin agonists [21], their combined effects on metabolic efficiency, oxidative balance, and vascular function may help stabilize circadian-metabolic interactions and indirectly support sleep–wake regulation [47,50].

Collectively, CBD, PUFAs, and sesame lignans influence sleep by converging across interconnected biological systems rather than acting through isolated pathways. Their complementary actions highlight the importance of integrated mechanisms across neurosensory, inflammatory, and neuromodulatory systems, which may have translational relevance for understanding and managing hyperarousal-related insomnia [13,16].

A systems-level synthesis of these interactions, highlighting their potential convergence across biological processes relevant to sleep regulation, is presented in Table 3.

## 9. Research Gaps and Directions for Future Mechanistic Inquiry

Work on cannabidiol (CBD), hemp seed oil (HSO), and black sesame oil (BSO) has expanded considerably, but the mechanistic picture remains incomplete and fragmented. Many findings derive from narrowly focused experiments conducted under heterogeneous conditions, limiting cross-study comparability and synthesis. As a result, it remains uncertain how molecular or cellular changes translate into measurable sleep-related outcomes. In particular, links between these compounds and vulnerability to insomnia remain only partially resolved. These effects may be mediated through inflammation, oxidative stress, altered arousal, metabolic disruption, or circadian instability.

A recurring weakness in the literature is that sleep is frequently treated as a secondary outcome or not measured directly. Although several biologically plausible pathways have been proposed based on laboratory data, relatively few studies evaluate sleep architecture, continuity, or arousal thresholds using standardized methodologies. This complicates interpretation, as improvements in upstream physiology do not necessarily translate into improvements in sleep, and observed effects may vary across insomnia phenotypes rather than reflect generalizable mechanisms.

Another unresolved issue concerns how these compounds might interact across shared biological systems. Cannabidiol (CBD), polyunsaturated fatty acids (PUFAs), and lignans each influence immune signaling, oxidative balance, metabolic regulation, and stress responsiveness, yet most studies consider these effects in isolation. To our knowledge, no empirical studies to date have directly investigated the combined administration or synergistic potential of all three compound categories together. Whether their actions converge, counteract one another, or depend on physiological context remains largely unknown. Integrated mechanistic models have been proposed to illustrate these potential synergies, as summarized in Table 2 and Table 3. However, empirical evidence at the integrated-system level—including limbic arousal circuitry, mitochondrial energetics, and circadian–metabolic coupling—remains fragmentary.

Dose, timing, and duration of exposure add further uncertainty. The biological effects of these compounds are unlikely to be uniform over time or across conditions and may vary according to circadian phase, metabolic state, stress reactivity, or dietary background. In addition to circadian rhythms, infradian biological cycles may influence sleep-related physiology. Cycles longer than 24 h, including menstrual and seasonal variations, can alter hormonal signaling, stress responsivity, and metabolic regulation, potentially modulating susceptibility to insomnia [19,52,53]. PUFA-related effects may depend on circadian lipid metabolism, whereas CBD appears particularly sensitive to baseline stress responsivity. In the absence of standardized dosing strategies or temporal profiling, optimal dose–response relationships in humans remain to be determined, making it difficult to determine when these interventions are most likely to meaningfully influence sleep-related physiology.

The translation of mechanistic findings into clinically relevant outcomes also remains underdeveloped. Only a limited number of studies combine objective sleep measures with biological markers such as inflammatory profiles, electrophysiological indices of arousal, or indicators of metabolic timing. Individual variability—including genetic differences in endocannabinoid signaling, fatty-acid metabolism, antioxidant capacity, and stress sensitivity—is rarely addressed, despite the well-recognized heterogeneity of insomnia. Uniform responses across individuals should therefore not be assumed. Importantly, different insomnia phenotypes—such as those driven primarily by neuroinflammation versus those characterized by autonomic hyperarousal—may respond uniquely to the neuroimmune or GABAergic pathways targeted by these compounds.

Advancing this field will require research designs that move beyond compartmentalized approaches. Integrative studies that combine molecular, metabolic, immune, and electrophysiological measures with direct assessments of sleep may help clarify which biological changes are most relevant to sleep–wake regulation. Such approaches will be essential for identifying when, and for whom, compounds such as CBD, PUFAs, and lignans are most likely to provide meaningful translational benefit.

## 10. Conclusions

This narrative review integrates mechanistic evidence across neurobiological, inflammatory, metabolic, and circadian domains to examine how cannabidiol (CBD), hemp seed oil (HSO), and black sesame oil (BSO) may influence upstream processes that shape sleep–wake regulation. Although these compounds have traditionally been examined within separate disciplinary frameworks, their bioactive components converge on several interconnected systems increasingly recognized as central to the pathophysiology of insomnia. These systems include excitatory–inhibitory balance within arousal circuitry, endocannabinoid tone, neuroimmune signaling, oxidative resilience, mitochondrial efficiency, and the coordination of circadian and metabolic rhythms.

Across these domains, a coherent mechanistic pattern emerges. CBD appears to modulate stress- and arousal-related neural systems, particularly through serotonergic and GABAergic mechanisms. In contrast, PUFAs derived from HSO primarily influence inflammatory resolution and metabolic processes relevant to sleep pressure and neural stability. Sesame-derived lignans from BSO contribute antioxidant and inhibitory support that may help preserve neurometabolic integrity.

Although each compound influences distinct biological processes, their effects converge on shared regulatory systems. These convergent actions may help modulate physiological hyperarousal and support sleep–wake stability.

At the same time, substantial uncertainties remain. Much of the evidence comes from molecular and preclinical work, with few studies directly linking these upstream processes to measurable changes in human sleep architecture. At present, dose–response relationships in humans remain to be determined. Furthermore, current findings do not establish definitive clinical efficacy or standardized therapeutic protocols.

Interactions among CBD, PUFAs, and lignans remain largely unexplored, and the influence of timing, metabolic state, and individual variability has not been adequately characterized. These gaps highlight the need for research approaches that integrate molecular mechanisms with translational biomarkers and clinically relevant sleep measures.

Taken together, this review provides a conceptual framework for considering natural compounds such as CBD, HSO, and BSO as modulators of biological systems implicated in insomnia. Rather than suppressing symptoms, these agents may modulate multiple pathways involved in sleep–wake regulation. Clarifying how these compounds interact across shared physiological systems may inform the development of targeted strategies—whether as standalone or adjunctive approaches—that support sleep–wake stability and long-term physiological resilience. These interpretations remain mechanistic and should not be taken as evidence of clinical efficacy or as a therapeutic recommendation.

## Figures and Tables

**Table 1:** Key bioactive components of cannabidiol (CBD), hemp seed oil, and black sesame oil and their major biological roles.

Source	Key Bioactive Components	Major Biological Roles	Relevance to Sleep-Related Biological Processes	References
Cannabidiol (CBD)	Cannabidiol	Endocannabinoid modulation; anti-inflammatory effects; regulation of stress-responsive neural circuits	May influence neurobiological processes associated with sleep–wake stability and stress-related arousal	[33,34]
Hemp Seed Oil (HSO)	Linoleic acid (LA); α-linolenic acid (ALA); γ-linolenic acid (GLA)	Anti-inflammatory effects; membrane lipid regulation; metabolic signaling	May contribute to metabolic–neuroimmune processes relevant to sleep regulation	[29,30,44]
Black Sesame Oil (BSO)	Sesamin; Sesamolin; Sesamol	Antioxidant activity; neuroprotective properties; modulation of lipid metabolism	May contribute to oxidative stress–related processes and neural stability relevant to sleep regulation	[18,31,45]

Abbreviations: CBD, cannabidiol; HSO, hemp seed oil; BSO, black sesame oil

**Table 2:** Mechanistic domains associated with cannabidiol (CBD), hemp seed oil-derived polyunsaturated fatty acids (PUFAs), and black sesame oil-derived lignans based primarily on preclinical evidence.

Mechanistic Domain	HSO (PUFAs)	CBD	BSO (Lignans)	Evidence Type	Key References
GABA-related signaling	Indirect membrane-mediated modulation of receptor environment	Context-dependent modulation of GABAergic transmission	Association with inhibitory signaling pathways	In vitro/animal (preclinical)	[24,31,35]
Serotonin-related pathways	No direct evidence	5-HT1A receptor involvement and serotonergic modulation	Indirect stress-related effects	Animal/preclinical	[39,40]
Endocannabinoid-related pathways	Precursor availability influencing lipid signaling	FAAH inhibition and modulation of endocannabinoid tone	Indirect metabolic and redox support	In vitro/animal (preclinical)	[29,55,56]
Inflammatory signaling	Lipid-mediated modulation of inflammatory pathways	Anti-inflammatory effects via cytokine regulation	Lignan-associated anti-inflammatory effects	In vitro/animal (preclinical)	[30,37,57]
Oxidative stress pathways	Reduction in lipid peroxidation	Antioxidant-associated effects	Direct antioxidant activity and redox modulation	In vitro/animal (preclinical)	[17,18,29]
Stress-related pathways	Support of metabolic and physiological resilience	Modulation of stress-responsive neural circuitry (HPA axis)	Endocrine buffering and stress-related modulation	In vitro/animal (preclinical)	[19,31,39]
Cellular membrane stability	Enhanced membrane fluidity and lipid environment	Indirect modulation of membrane-associated signaling	Stabilization of cellular membrane structures	In vitro/experimental (preclinical)	[29,58]

Note: This table summarizes mechanistic associations derived primarily from preclinical and experimental studies. These pathways indicate biological plausibility and do not imply direct effects on sleep outcomes or clinical efficacy. Abbreviations: CBD, cannabidiol; HSO, hemp seed oil; BSO, black sesame oil; PUFAs, polyunsaturated fatty acids; GABA, gamma-aminobutyric acid; FAAH, Fatty Acid Amide Hydrolase; HPA, hypothalamic–pituitary–adrenal; 5-HT1A, 5-hydroxytryptamine receptor 1A.

**Table 3:** Systems-level integration of mechanistic pathways linking cannabidiol (CBD), hemp seed oil-derived polyunsaturated fatty acids (PUFAs), and black sesame oil-derived lignans to biological domains relevant to hyperarousal-related insomnia.

Biological Domain	CBD	HSO (PUFAs)	BSO (Lignans)	Integrated Interpretation	Evidence Type	Supporting References
Arousal regulation	Attenuation of stress-related neural activity via serotonergic and endocannabinoid pathways	Support of metabolic homeostasis	Indirect anxiolytic and antioxidant-effects	Reduced hyperarousal and improved system stability	Preclinical	[31,35,39]
Excitatory–inhibitory balance	Context-dependent modulation of inhibitory signaling (GABAergic pathways)	Modulation of membrane stabilization affecting receptor function	Association with inhibitory signaling and neuronal stability	Reduced cortical excitability	Preclinical	[24,40,54]
Stress-responsive neurocircuitry	Modulation of limbic activity and HPA-axis signaling	Support of metabolic and vascular function	Endocrine buffering and stress-related modulation	Dampened stress–arousal coupling	Preclinical	[12,19,41]
Neuroimmune regulation	Anti-inflammatory effects via cytokine modulation	Lipid-mediated inflammatory resolution	Antioxidant and anti-inflammatory effects	Improved immune–arousal balance	Preclinical	[30,37,57]
Oxidative and mitochondrial resilience	Reduction in oxidative stress and support of cellular homeostasis	Mitochondrial support and lipid metabolism	Protection against lipid peroxidation and oxidative damage	Enhanced neurometabolic stability	Preclinical	[18,29,50]
Circadian–metabolic coordination	Indirect modulation of rhythmic activity via ECS pathways	Metabolic regulation and lipid signaling	Redox-associated support	Improved rhythmic–metabolic alignment	Conceptual/Preclinical	[21,50,51]

Note: This table presents a conceptual systems-level synthesis of convergent mechanisms relevant to sleep regulation. The proposed interactions are based primarily on preclinical and experimental evidence and reflect biological plausibility rather than validated causal relationships. These interactions should not be interpreted as confirmed synergistic effects on sleep architecture or clinical outcomes. Abbreviations: CBD, cannabidiol; HSO, hemp seed oil; BSO, black sesame oil; PUFAs, polyunsaturated fatty acids; HPA, hypothalamic–pituitary–adrenal; ECS, endocannabinoid system.

## Data Availability

No new data were created or analyzed in this study. Data sharing is not applicable to this article.
